# A Comparative Study of Systolic and Diastolic Mechanical Synchrony in Canine, Primate, and Healthy and Failing Human Hearts

**DOI:** 10.3389/fcvm.2021.750067

**Published:** 2021-10-28

**Authors:** Tiangang Zhu, Ming Lei, Zhilong Wang, Rongli Zhang, Yan Zhang, Wenying Jin, Chao Yu, Christopher L.-H. Huang, Dongyue Liu, Wen Zheng, Yuli Liu, Xin Quan, Lingyun Kong, Siying Liang, Xiuqin Zhang

**Affiliations:** ^1^Department of Cardiology, Peking University People's Hospital, Beijing, China; ^2^Medical Sciences Division, Department of Pharmacology, University of Oxford, Oxford, United Kingdom; ^3^Key Laboratory of Medical Electrophysiology of Ministry of Education, Institute of Cardiovascular Research, Southwest Medical University, Luzhou, China; ^4^Department of Cardiology, The Eighth Affiliated Hospital, Sun Yat-sen University, Shenzhen, China; ^5^Institute of Molecular Medicine, College of Future Technology, Peking University, Beijing, China; ^6^Case Cardiovascular Research Institute, Institute for Molecular Transformative Medicine, Case Western Reserve University, Cleveland, OH, United States; ^7^Beijing Key Laboratory of Cardiometabolic Molecular Medicine, Peking University, Beijing, China; ^8^Physiological Laboratory, University of Cambridge, Cambridge, United Kingdom; ^9^Department of Biochemistry, University of Cambridge, Cambridge, United Kingdom; ^10^Department of Cardiology, National Center for Cardiovascular Diseases, Fuwai Hospital, Beijing, China; ^11^Beijing Tsinghua Changgung Hospital, Beijing, China

**Keywords:** mechanical synchrony, interventricular mechanical delays (IVMD), systolic interventricular mechanical delays (IVMDs), diastolic interventricular mechanical delays (IVMDd), pulsed-wave Doppler echocardiography

## Abstract

**Aim:** Mechanical dyssynchrony (MD) is associated with heart failure (HF) and may be prognostically important in cardiac resynchronization therapy (CRT). Yet, little is known about its patterns in healthy or diseased hearts. We here investigate and compare systolic and diastolic MD in both right (RV) and left ventricles (LV) of canine, primate and healthy and failing human hearts.

**Methods and Results:** RV and LV mechanical function were examined by pulse-wave Doppler in 15 beagle dogs, 59 rhesus monkeys, 100 healthy human subjects and 39 heart failure (HF) patients. This measured RV and LV pre-ejection periods (RVPEP and LVPEP) and diastolic opening times (Q-TVE and Q-MVE). The occurrence of right (RVMDs) and left ventricular systolic mechanical delay (LVMDs) was assessed by comparing RVPEP and LVPEP values. That of right (RVMDd) and left ventricular diastolic mechanical delay (LVMDd) was assessed from the corresponding diastolic opening times (Q-TVE and Q-MVE). These situations were quantified by values of interventricular systolic (IVMDs) and diastolic mechanical delays (IVMDd), represented as positive if the relevant RV mechanical events preceded those in the LV. Healthy hearts in all species examined showed greater LV than RV delay times and therefore positive IVMDs and IVMDd. In contrast a greater proportion of the HF patients showed both markedly increased IVMDs and negative IVMDd, with diastolic mechanical asynchrony negatively correlated with LVEF.

**Conclusion:** The present IVMDs and IVMDd findings have potential clinical implications particularly for personalized setting of parameter values in CRT in individual patients to achieve effective treatment of HF.

## Introduction

Heart failure (HF), a condition associated with high mortality and morbidity, constitutes a major and growing worldwide public health problem (1–4), resulting in a requirement for the development of effective management. To this end, cardiac resynchronization therapy (CRT) implantation has proved useful as a therapeutic strategy for managing HF (5, 6). However, 30% of such treated patients fail to show beneficial outcomes (7) or even show further deterioration in cardiac mechanics and function with CRT implantation (8–10). CRT likely addresses the association of HF with mechanical dyssynchrony (MD) or disparities in wall contraction timing (8). This may therefore reflect the importance of variations in the contributory factors for HF in individual patients. These include delayed electrical conduction or electromechanical coupling, or altered regional myocardial properties following ischemic damage or myocardial infarction (8). Previous experimental reports had positively correlated durations of canine ventricular depolarization and repolarization intervals with wall thickness and cardiac size. The latter potentially alter in HF (11) causing both pro-arrhythmic effects (12, 13) and mechanical dyssynchrony. However, contributions of electromechanical coupling are less well understood.

Nevertheless, left ventricular mechanical delays (LVMDs), assessed using ultrasound methods (14) are useful prognosticators in both ischemic (15) and nonischemic dilated cardiomyopathy (16), acute myocardial infarction (17) and coronary artery disease (18). They may also be of prognostic importance following CRT implantation (7).

However, to this end, little is known about patterns of either normal or abnormal ventricular mechanical synchronization. This applies to both the right or left ventricle, whether to systolic or diastolic function, or to disparities between them, between different species of large mammals, or between healthy and diseased human hearts. Previous studies using M-mode, pulse-wave Doppler and tissue Doppler imaging have been confined to human studies of systolic as opposed to diastolic, and left ventricular asynchrony in HF patients (19–23). In particular, few studies to date have explored their diastolic ventricular asynchrony.

The present study therefore extends these previous studies in the following respects for the first time. We examine diastolic in addition to systolic ventricular synchrony. We also provide simultaneous readouts from the right in addition to the left ventricle. Such measures are surveyed and compared in exemplars of large mammalian canine and nonhuman primate species, of potential value in future experimental studies, in addition to human hearts. Finally the analyses are extended to HF patients. We thus characterize and compare left and right ventricular, systolic and diastolic mechanical sequences in canine, primate, and normal and failing human hearts for the first time.

We accordingly applied clinically accepted diagnostic electrocardiographic and echocardiographic methods to obtain and compare: (a) As systolic indicators: right and left ventricular pre-ejection periods (RVPEP and LVPEP) and (b) As diastolic indicators: mitral and tricuspid diastolic opening times (Q-MVE and Q-TVE). Corresponding values from these measurements were also compared between the right and left ventricles, between normal hearts in the species examined and in failing human hearts. This additionally provided incidences of the situations where there were relative right or left ventricular delays as well as the resulting interventricular, systolic or diastolic delays.

We have demonstrated for the first time that (a) normal animal and human hearts show greater left than right delay times and therefore positive systolic (IVMDs) and diastolic IVMD delays (IVMDd). (b) In contrast, a greater proportion of individual HF patients show reversals in both these IVMDs and IVMDd trends.

Our demonstration of such left/right mechanical differences and variations in patterns of both systolic and diastolic function between individual hearts thus have potential implications for clinical therapeutic techniques based on correcting mechanical activation times such as cardiac resynchronization therapy. Thus, the findings suggest that systolic and diastolic ventricular mechanical sequence assessment by echocardiography should be performed before and after CRT, in individual patients as a guide for the optimization of pacing indices. Furthermore, effectiveness of the therapeutic response may be optimized with the aid of determinations of ventricular mechanical sequence in individual patients and monitored by echocardiographic examinations.

## Methods

### Study Populations

The experimental studies were performed on fifty-nine rhesus monkeys (age 15.5 ± 3.1 years) and fifteen beagle dogs (age 2 years). The animals were anesthetized (14 mg/kg ketamine for monkeys; 1–2% isoflurane for dogs) for echocardiography. All procedures were approved by the Animal Care and Use Committee of Peking University (2011-0010) and complied with the principles of laboratory animal care of the National Academy of Sciences/National Research Council of the People's Republic of China. The clinical studies were performed on 100 healthy subjects (range: 19–56 years; mean age: 29.7 ± 7.18 years, 50 males, 50 females) and 39 patients with systolic heart failure (age range 31–80 years; mean age: 57.1 ± 17.2 years, 30 males). All human subjects provided clinical histories and underwent physical, electrocardiogram and transthoracic echocardiographic examination. This study was approved by the local Ethics Committee and the informed consents were obtained from all subjects.

The inclusion criteria for normal subjects included: (1) normal electrocardiogram; (2) normal values in all echocardiographic measurements; left ventricular ejection fraction (LVEF) ≥ 55%, Peak velocity of mitral valve measured by Pulse wave Doppler / Peak velocity of mitral annulus measured by tissue Doppler in early diastolic of left ventricle (E/e') <15 for the septum and E/e' <13 for the lateral wall; (3) absence of any history of cardiovascular disease including hypertension, coronary, myocardial, diabetic and thyroid disease; (4) close to ideal echocardiographic image quality. The inclusion criteria for the HF patients were: (1) clinical diagnosis of heart failure with New York Heart Association (NYHA) Functional Classification grades II~IV; (2) LVEF derived using Bi-plane Simpson's rule ≤ 45%; (3) electrocardiographic sinus rhythm with QRS complex <120 ms in duration with no evidence of conduction abnormality; (4) no echocardiographic evidence for valvular disease; (5) no history of thoracotomy operations; (6) close to ideal echocardiographic image quality.

### Echocardiographic Measurements

**Figure 1:**
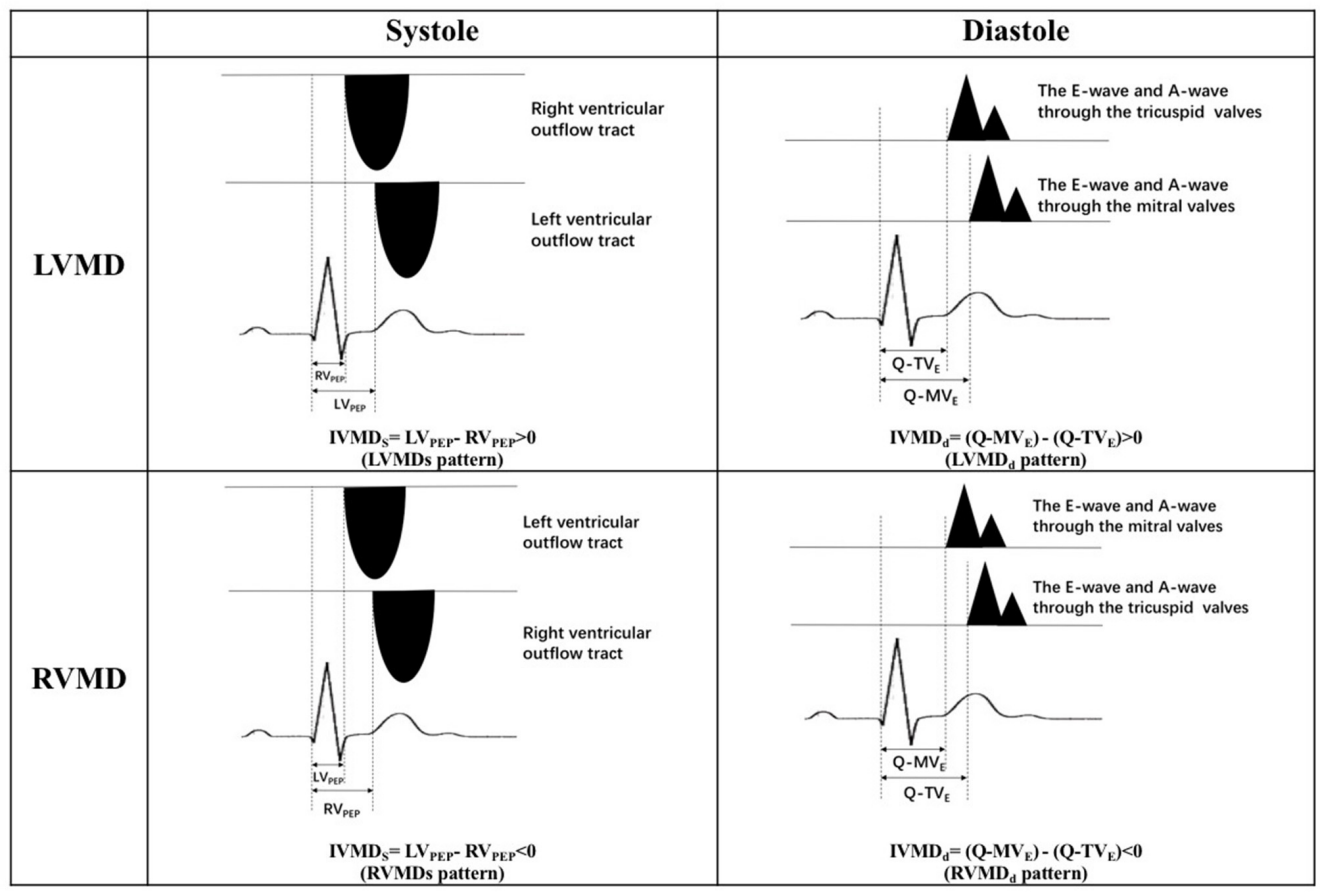
Four mechanical delay patterns. LVMDs, left ventricular mechanical delay in systole; RVMDs, right ventricular mechanical delay in systole; LVMDd, left ventricular mechanical delay in diastole; RVMDd, right ventricular mechanical delay in diastole; IVMDs, systolic interventricular mechanical delay; IVMDd, diastolic interventricular mechanical delay; LV_PEP_, left ventricular pre-ejection period; RV_PEP_, right ventricular pre-ejection period; Q-MVe, the time interval from the onset of QRS complex to the onset of the early diastolic E wave of the mitral valve; Q-TVe, the time interval from the onset of QRS complex to the onset of the early diastolic E wave of the tricuspid valve.

**Figure 2:**
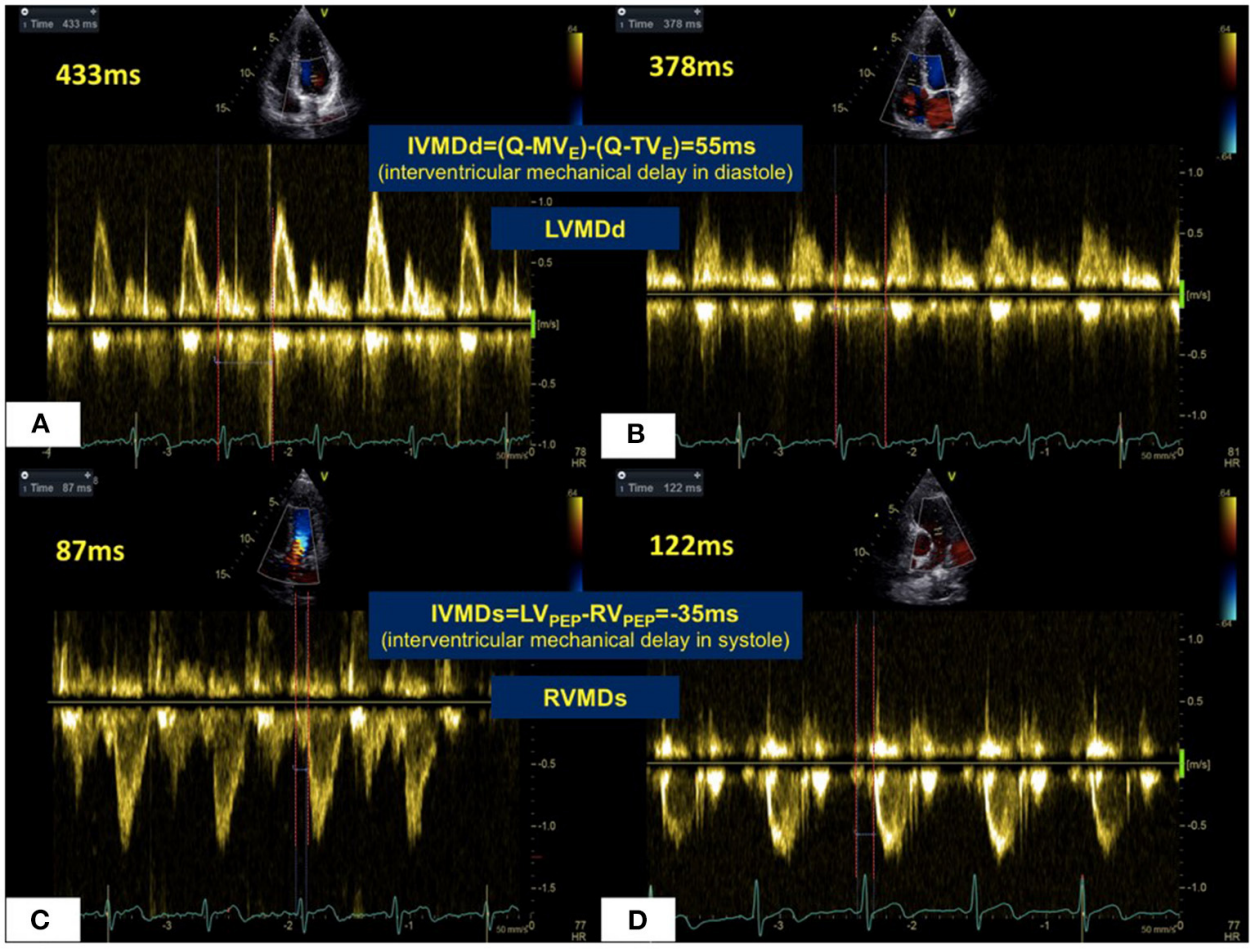
Mechanical sequences in the LV and RV in a normal healthy subject. In diastole **(A,B)**, RV filling occurred 55 ms prior to LV filling giving a LVMDd pattern. For systolic ejection **(C,D)**, LV ejection preceded RV ejection by 35 ms in RVMDs pattern. Abbreviations as in legend to Figure 1.

**Figure 3:**
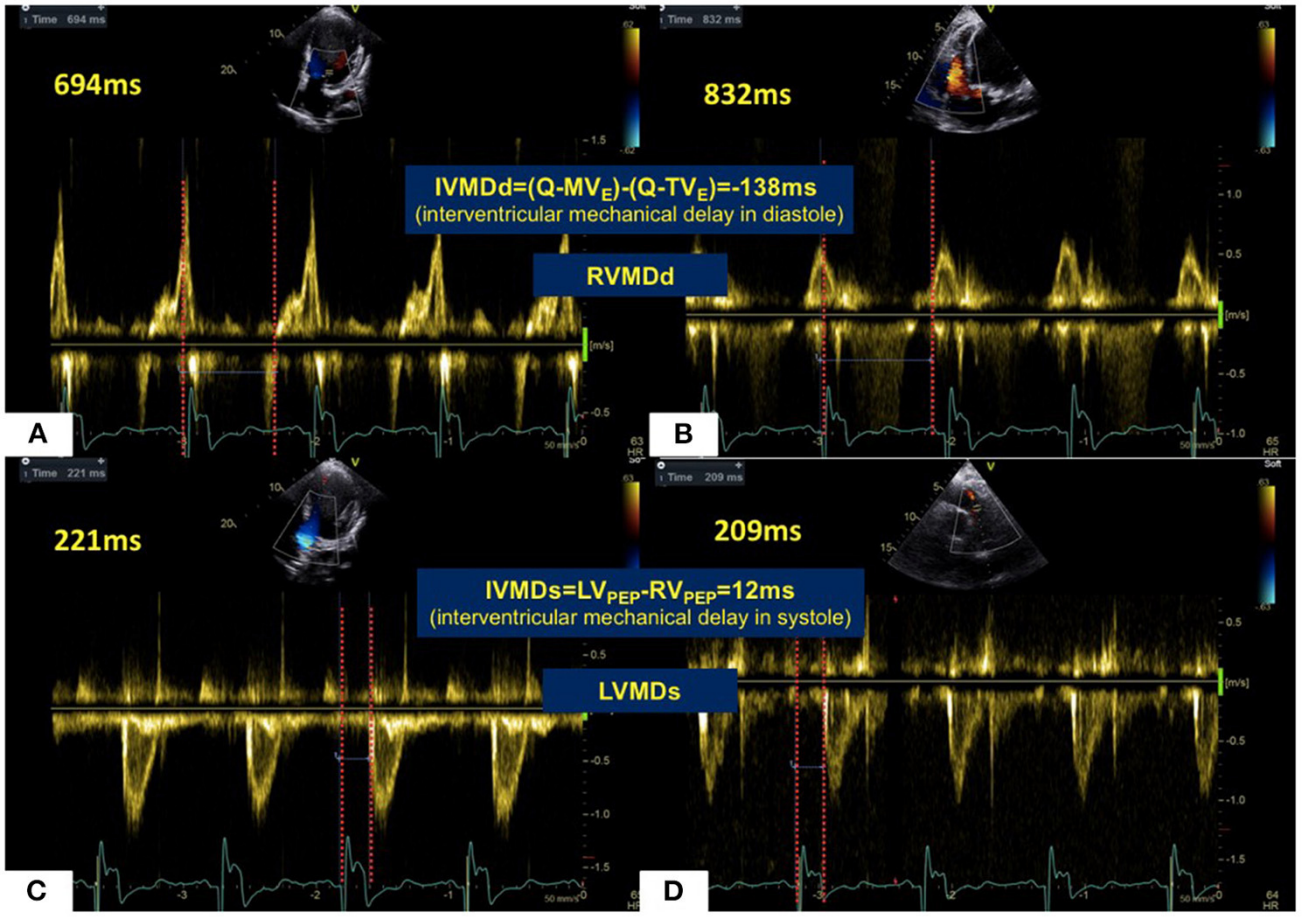
Mechanical sequences between LV and RV in a patient with HF. In diastole **(A,B)**, LV filling occurred 138 ms prior to RV filling giving a RVMDd pattern. This was in marked contrast to most healthy subjects. For systolic ejection **(C,D)**, RV ejection preceded LV ejection by 12 ms in a LVMDs pattern. Abbreviations as in legend to Figure 1.

Transthoracic echocardiographic examinations were performed in all subjects (GE Vivid 7 for animal studies, Vivid E9, GE Vingmed, Horten, Norway; ALOKA Prosound F75, Tokyo, Japan, for human studies with a 3–6 MHz phased array transducer). Standard two-dimensional echocardiography with Doppler examination was performed according to American Society of Echocardiography guidelines (24). All acquired images were stored for three consecutive cardiac cycles. The spectrum of Pulse wave Doppler for the left ventricular outflow tract (LVOT) was obtained from the apical five-chamber views, and that for the right ventricular outflow tract (RVOT) was obtained from the pulmonary long-axis views using pulsed-wave Doppler echocardiography. Left ventricular pre-ejection period (LV_PEP_) was measured as the interval from the beginning of the QRS complex to the onset of aortic valve opening. The right ventricular pre-ejection period (RV_PEP_) was measured as the interval from the beginning of the QRS complex to the onset of pulmonary valve opening. Transmitral and transtricuspid inflow Doppler wave patterns were respectively recorded in apical five-chamber views. The diastolic opening time of the left and right ventricles was measured as the interval between the onset of the QRS complex and the beginning of the E wave for the mitral valve (Q-MV_E_) and for the tricuspid valve (Q-TV_E_) respectively. Four mechanical delay patterns are shown in Figure 1. The occurrence of a left ventricular mechanical delay in systole (LVMDs) was identified when RV_PEP_ < LV_PEP_. Conversely, the existence of a right ventricular mechanical delay in systole (RVMDs) was identified when RV_PEP_ > LV_PEP_ (Figure 2). Similarly, the existence of a left ventricular mechanical delay in diastole (LVMDd) was identified when Q-MVe > Q-TVe, and the existence of a right ventricular mechanical delay in diastole (RVMDd) was identified when Q-MVe < Q-TVe (Figure 3). The interventricular mechanical delay (IVMD) was defined as the interval between the LV and RV mechanical delays. IVMD was defined as positive if RV activation preceded the LV activation and negative if LV activation preceded RV activation.

### Statistical Analysis

Continuous variables were expressed as mean ± SD and compared between two groups by *t* test for independent samples. When not normally distributed, continuous data was expressed as median (± the interquartile range, IQR) and compared between two groups by the non-parametric Mann-Whitney test. Categorical variables were expressed as percentages and compared between two groups by χ^2^ testing. Linear regression was performed to compare values of IVMDs, IVMDd and LVEF in the HF patients. Binary logistic regression analyses were performed to determine the influencing factors on the ventricular mechanical sequence. All tests were two-tailed and *p*-values were assessed with a significance level of 0.05. All statistical analyses were performed using SPSS version 21.0 (IBM Corporation, Armonk, NY, USA).

## Results

### Baseline Characteristics

Table 1 lists overall baseline characteristics for all the study subjects. All animals were males with normal echocardiographic ejection fractions. One hundred healthy subjects (Age: 19–56 years; mean age: 29.7 ± 7.18 (SD) y, 50 males) and 39 patients with HF (Age: 31–80 years; mean: 57.1 ± 17.2 (SD) years, 30 males) were studied. The healthy subjects were much younger than the patients owing to the strict study inclusion criteria.

**Table 1:** Baseline characteristic of the study subjects.

	**Animals**		
	**Primates (*n* = 59)**	**Canines (*n* = 15)**	***p* Value**
Age (year)	15.5 ± 3.09	2.1 ± 0.03	<0.001
BW (kg)	13.8 ± 4.03	13.36 ± 3.93	0.325
Male (%)	100	100	1.000
Heart rate (bpm)	129.0 ± 26.1	114.2 ± 43.3	0.018
SBP (mmHg)	149.0 ± 24.0	65.3 ± 17.9	<0.001
DBP (mmHg)	80.4 ± 15.9	48.4 ± 16.6	<0.001
LVEF (%)	70.1 ± 5.7	59.8 ± 9.3	<0.001
	**Humans subject**		
	**Normal (*****n*** **=** **100)**	**HF (*****n*** **=** **39)**	***p*** **Value**
Age (year)	29.7 ± 7.2	57.1 ± 17.2	<0.001
Male (%)	50.0	76.9	<0.001
Heart rate (bpm)	67.1 ± 8.4	77.2 ± 16.4	0.056
BSA (m^2^)	1.7 ± 0.2	1.8 ± 0.2	0.568
LVEF (%)	64.8 ± 4.8	33.8 ± 7.2	<0.001
LVEDV (ml)	101.5 ± 24.2	204.0 ± 86.9	<0.001
LVESV (ml)	35.8 ± 10.3	146.4 ± 59.1	<0.001
E/e' (sep)	6.7 ± 1.1	21.1 ± 26.9	<0.001
E/e' (lat)	4.8 ± 0.9	12.4 ± 7.1	<0.001
RV-S' (m/s)	0.15 ± 0.02	0.12 ± 0.03	0.329
QRS duration (ms)	92.6 ± 9.1	105.6 ± 13.5	0.102
PR (ms)	146.0 ± 11.5	150.0 ± 40.0	0.168
QTc (ms)	415.0 ± 18.5	499.6 ± 24.2	<0.001

*Data are represented as mean ± SD*.

*HF, heart failure; BSA, body surface area; BW, body weight; DBP, diastolic blood pressure; FAC, fractional area change; LVEF, left ventricular ejection fraction; LVEDV, left ventricular end-diastolic volume; LVESV, left ventricular end-systolic volume; RV, right ventricle; SBP, systolic blood pressure*.

### Ventricular Mechanical Delays

Tables 2, 3 summarize the Doppler echocardiographic measurements of the mechanical delays in left and right ventricular contraction and relaxation. The healthy canine, monkey and human hearts showed differing ventricular mechanical delays (Table 2). Nevertheless, the ventricular diastolic delay time (Q-MV_E_, Q-TV_E_) was greater than the systolic delay time (LV_PEP_, RV_PEP_), giving greater diastolic than systolic interventricular mechanical delays (IVMD). The human studies went on to demonstrate that HF patients showed greater RV and LV mechanical delays than normal subjects in systole and diastole. Furthermore, patients with HF showed significantly reduced or even negative IVMDd, suggesting a proportionally greater increase in RVMDd compared to normal subjects (Table 3).

**Table 2:** Species comparison of ventricular mechanical delays in healthy hearts.

	**Canines**	**Primates**	**Humans**	***p* value**
	**(*n* = 15)**	**(*n* = 59)**	**(*n* = 100)**	
LV_PEP_ (ms)	58.3 ± 18.8	43.6 ± 8.8	46.8 ± 12.4	<0.001
RV_PEP_ (ms)	47.7 ± 13.6	42.0 ± 8.8	40.3 ± 11.1	0.041
Q-MVe (ms)	297.9 ± 31.1	282.7 ± 33.8	396.8 ± 33.0	<0.001
Q-TVe (ms)	258.0 ± 35.3	253.8 ± 35.3	379.1 ± 33.1	<0.001
IVMDs (ms)	7.4 ± 12.8	1.6 ± 6.5	6.5 ± 1.3	<0.001
IVMDd (ms)	38.6 ± 26.7	28.9 ± 13.6	17.7 ± 0.1	<0.001

*Data are represented as mean ± SD*.

*LV_PEP_, left ventricular pre-ejection period; RV_PEP_, right ventricular pre-ejection period; Q-MVe, time interval from the onset of QRS complex to the onset of early diastolic E wave of mitral valve; Q-TVe, time interval from the onset of QRS complex to the onset of early diastolic E wave of tricuspid valve; IVMDs, systolic interventricular mechanical delay; IVMDd, diastolic interventricular mechanical delay*.

**Table 3:** Ventricular mechanical delays compared in normal and failing human hearts.

	**Normal**	**HF**	***p* value**
	**(*n* = 100)**	**(*n* = 39)**	
LV_PEP_ (ms)	46.8 ± 12.4	92.3 ± 29.9	<0.001
RV_PEP_ (ms)	40.3 ± 11.1	75.8 ± 25.4	<0.001
Q-MVe (ms)	396.8 ± 33.0	421.2 ± 54.6	<0.001
Q-TV_E_ (ms)	379.1 ± 33.1	430.3 ± 54	<0.001
IVMDs (ms)	6.5 ± 1.3	16.5 ± 20.6	<0.001
IVMDd (ms)	17.7 ± 0.1	−9.1 ± 54.1	<0.001

*HF, heart failure*.

*Other abbreviations as in legend to Table 2*.

**Figure 4:**
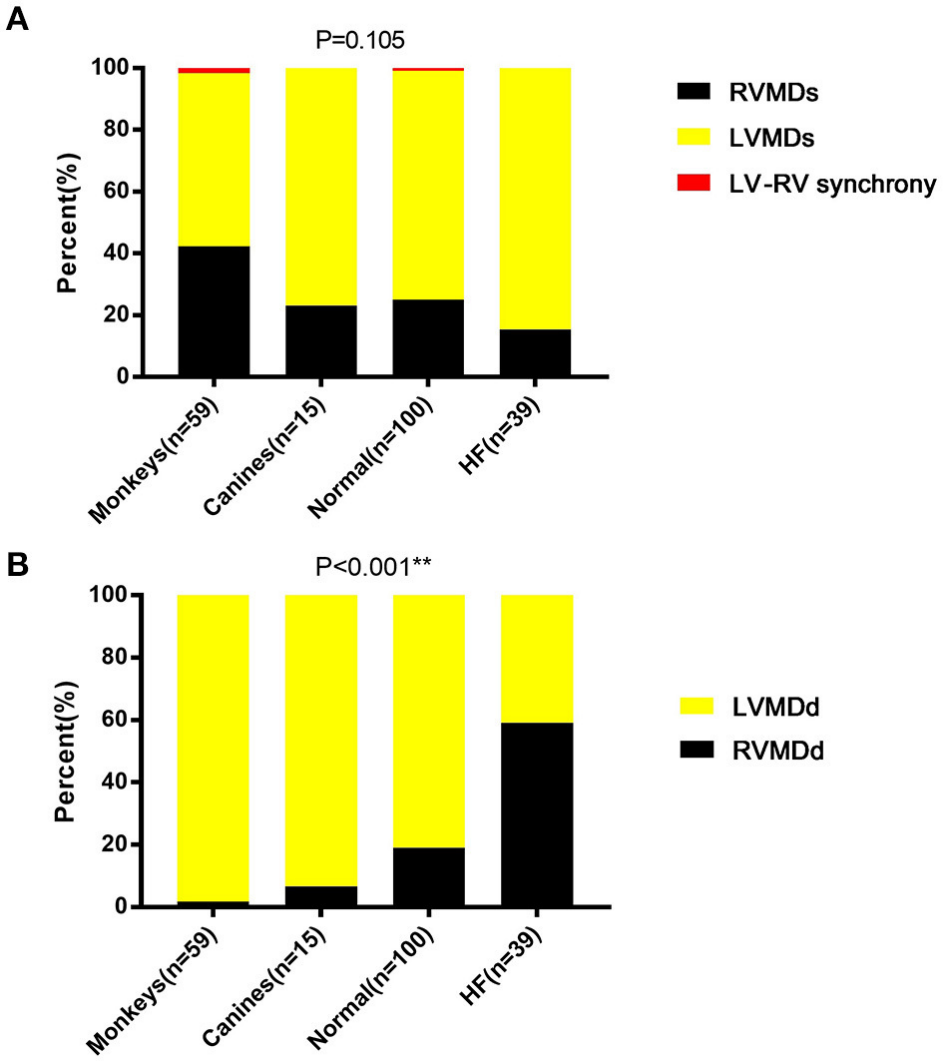
Ventricular mechanical sequences in **(A)** systole and **(B)** diastole. LVMDs, left ventricular mechanical delay in systole; LVMDd, left ventricular mechanical delay in diastole; RVMDs, right ventricular mechanical delay in systole; RVMDd, right ventricular mechanical delay in diastole. **denotes results satisfying the significance criterion *p* < 0.01.

### Ventricular Mechanical Sequences

To compare differences in mechanical sequence patterns between LV and RV, echocardiographic parameters related to systole or diastole were assessed in each individual experimental or human subject. The percentage occurrences of LVMDs, RVMDs, LVMDd and RVMDd were then determined (Tables 4, 5). The percentage occurrences of LVMDs in normal canine, primate and human hearts was 76.9% (95%CI, 0.54–1.0), 55.9% (95%CI, 0.43–0.69), and 74.0% (95%CI, 0.65–0.83) respectively. That for patients with HF was 84.6%, (95%CI, 0.73–0.96). The corresponding percentage occurrences of LVMDd was 93.3% (95%CI, 0.81–1.0), 98.3% (95%CI, 0.95–1.0) and 81.0% (95%CI, 0.73–0.89). In contrast, that for patients with HF was only 41.1% (95%CI, 0.26–0.56). Figures 3A,B summarizes the occurrences for LVMDs and RVMDs, and LVMDd and RVMDd respectively, illustrating the highly consistent tendency for a later RV filling in the patients with HF. Finally, binary logistic regression analyses did show associations with variations in the ventricular mechanical sequence like age, sex, BSA, and heart rate (*p* > 0.05) (Supplementary Materials).

**Table 4:** Comparative analysis of the ventricular mechanical sequence in healthy hearts.

	**Canines**	**Primates**	**Humans**	***P* value**
	**(*n* = 15)**	**(*n* = 59)**	**(*n* = 100)**	
**Systolic mechanical sequence [% (95% CI)]**				
RVMDs	23.1 (0–46)	42.4 (30–55)	25.0 (17–33)	0.003
LVMDs	76.9 (54–100)	55.9 (43–69)	74.0 (65–83)	0.365
LV-RV synchrony	-	1.7 (0–5)	1.0 (0–3)	0.638
**Diastolic mechanical sequence [% (95% CI)]**				
RVMDd	6.7 (0–19)	1.7 (0–5)	19.0 (11–27)	0.103
LVMDd	93.3 (81–100)	98.3 (95–100)	81.0 (73–89)	0.368
LV-RV synchrony	-	-	-	

*CI, confidence intervals; LV, left ventricle; RV, right ventricle; LVMDs, left ventricular mechanical delay in systole; LVMDd, left ventricular mechanical delay in diastole; RVMDs, right ventricular mechanical delay in systole; RVMDd, right ventricular mechanical delay in diastole*.

**Table 5:** Analysis of the ventricular mechanical sequence- normal humans vs HF patients.

	**Normal**	**HF**	***P* value**
	**(*n* = 100)**	**(*n* = 39)**	
**Systolic mechanical sequence [% (95%CI)]**			
RVMDs	25.0 (17–33)	15.4 (4–27)	0.168
LVMDs	74.0 (65–83)	84.6 (73–96)	0.365
LV-RV synchrony	1.0 (0–3)	-	
**Diastolic mechanical sequence [% (95%CI)]**			
RVMDd	19.0 (11–27)	59.0(44–74)	<0.001
LVMDd	81.0 (73−89)	41.0(26–56)	<0.001
LV-RV synchrony	-	-	

*HF, heart failure*.

*Other abbreviations as in legend to Table 4*.

### Correlations Between IVMDs and IVMDd and LVEF in HF Patients

**Figure 5:**
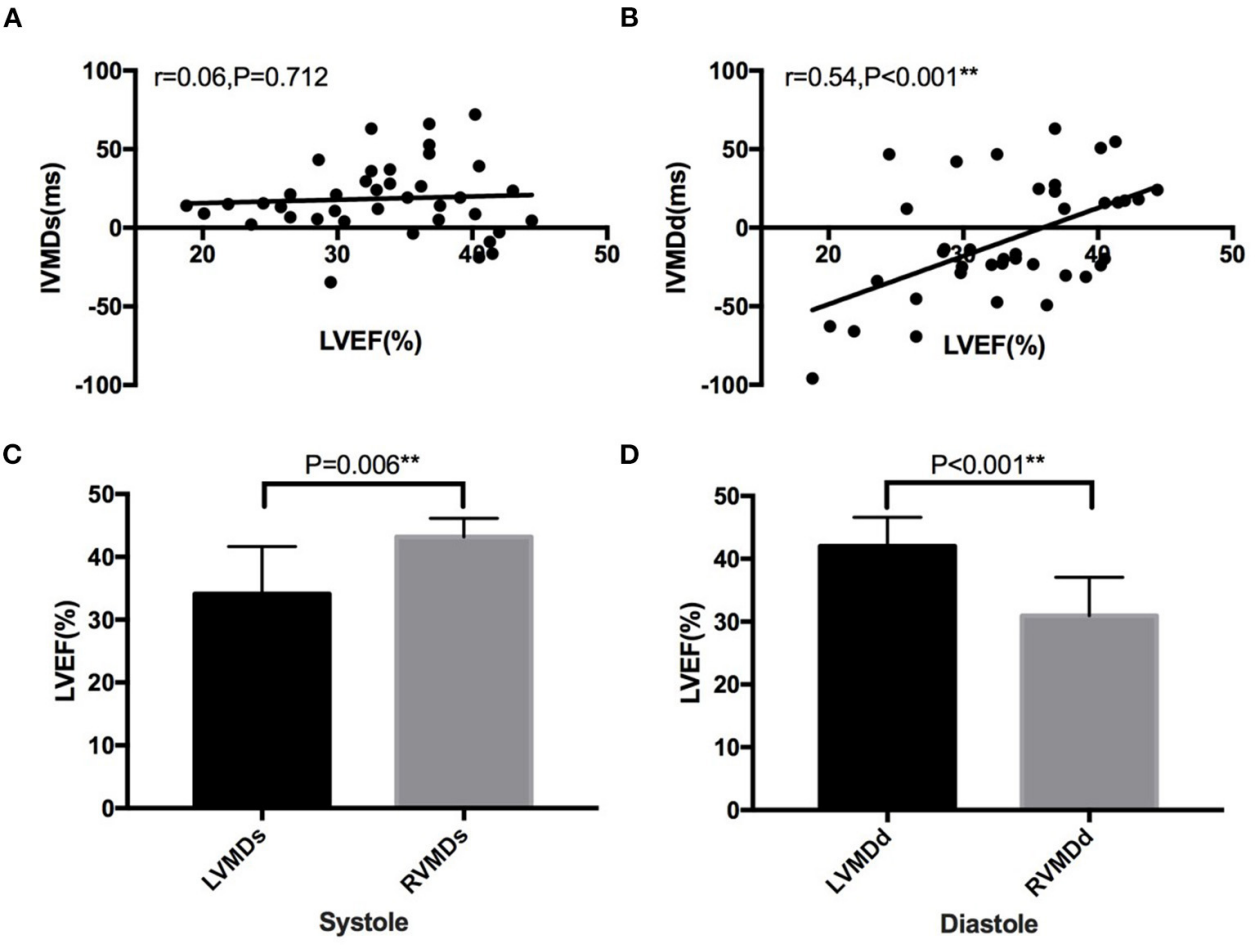
Correlation between IVMD and LVEF in patients with heart failure. No significant correlation between **(A)** IVMDs and LVEF (*r* = 0.06, *p* = 0.712), but a positive correlation between **(B)** IVMDd and LVEF (*r* = 0.54, *p* < 0.001). Significant differences in LVEF between HF patients with different ventricular mechanical sequences **(C,D)**. LVMDs, left ventricular mechanical delay in systole; LVMDd, left ventricular mechanical delay in diastole; RVMDs, right ventricular mechanical delay in systole; RVMDd, right ventricular mechanical delay in diastole; IVMD, interventricular mechanical delay; IVMDs, systolic interventricular mechanical delay; IVMDd, diastolic interventricular mechanical delay; LVEF, left ventricular ejection fraction. **denotes results satisfying the significance criterion *p* < 0.01.

Linear regression was performed to compare IVMDs/IVMDd and LVEF in the patients with HF (Figure 5). This demonstrated no significant correlation between IVMDs and LVEF (*r* = 0.06, *p* = 0.712), but a positive correlation between IVMDd and LVEF (*r* = 0.54, *p* < 0.001). The greater the negative value of IVMDd (RVMDd pattern), the lower the LVEF. Furthermore, HF showed differing occurrences in their patterns of particular systolic and diastolic characteristics. A greater proportion of HF patients showed associations between LVEF and RVMDs, than between LVEF and LVMDs (43.2 ± 1.21 vs. 34.1 ± 1.32%, *p* = 0.006). In contrast, HF patients showed less marked associations between LVEF and RVMDd than between LVEF and LVMDd (31.0 ± 1.27 vs. 42.0 ± 1.15%, *p* < 0.001).

## Discussion

We performed comparative echocardiographic analyses of ventricular mechanical activation sequences bearing on the synchrony of left and right ventricular systole and diastole in experimental animals, normal human subjects and HF patients. We report a predominance of left ventricular mechanical delay in systole (LVMDs) (76.9, 55.9, and 74.0% respectively) and highly consistent diastolic filling patterns (LVMDd) (93.3, 98.3, and 81.0% respectively) on the basis of paired comparisons of LV and RV parameters in each subject, in normal canine, primate and human hearts. Previous reports (21, 22) had not detected deviations from synchronous or slightly earlier LV relative to RV mechanical activation on the basis of their unpaired comparisons of mean LV and RV ejection time through entire experimental groups. The later LV repolarization times observed here may reflect the thicker LV walls resulting in prolonged LV repolarization times. Previous animal studies have positively correlated such repolarization times with wall thickness (25, 26). Our present observation of significantly reduced LVMDd to 41.1% in HF patients, could then reflect changes in LV wall anatomy and electrical conduction. This would be consistent with our findings that the negative value of IVMDd was positively associated with the reduction in LVEF (Figure 5). IVMDd may thus have potential prognostic significance in HF.

The present echocardiographic ventricular mechanical sequence findings have potential implications for therapeutic cardiac resynchronization therapy (CRT), with applicability to assessment and optimization of CRT. First, current bi-ventricular pacing in CRT typically sets the LV pacing time to be simultaneous with or slightly later than that of the RV. However, we here report differing ventricular mechanical sequences between individuals which could then confound the effectiveness of CRT. Furthermore, HF patients showed altered RV and LV mechanical sequences with potential implications for CRT optimization. Such differences in RV and LV systolic and diastolic sequences may require transthoracic echocardiographic determination for optimization and monitoring of CRT.

Secondly, prolonged QRS duration is currently used to measure ventricular mechanical dyssynchrony as an indication for CRT in HF. However, electrical dyssynchrony may not exactly parallel mechanical dyssynchrony. Thus, Yu et al. (27) reported that LV systolic and diastolic mechanical asynchrony are common in HF patients with normal QRS durations. Significant systolic asynchrony was thus observed in >40% of patients with narrow QRS complexes. Conversely, ~36% of HF patients with wide QRS complexes (>120 ms) did not have obvious intraventricular asynchrony. Furthermore, there was no correlation between the degree of LV asynchrony and QRS duration. Gabe B et al. reported a similar result (28). Furthermore, baseline QRS duration was not a good predictor for a better response to CRT in HF patients. Neither baseline nor shortening of QRS duration were good predictors for hemodynamic, clinical or echocardiographic improvement. However, the baseline severity of ventricular dyssynchrony assessed by echocardiography proved of predictive value (29, 30).

Thirdly, assessment of interventricular dyssynchrony may be critical for optimizing interventricular delays (V-V delays) in CRT. Their optimal timing improves ventricular filling capacity and stroke volume, reducing mitral regurgitation and reversing LV remodeling with subsequent reductions in short-term morbidity and mortality. Admittedly, there is evidence for intraventricular but not interventricular synchrony as an independent prognostic indicator in CRT patients. However, previous work had assessed IVMDs as a predictor without considering the interventricular mechanical sequence with V-V delays, setting LV timing simultaneously with or slightly earlier than RV. This contrasts with the LVMDs patterns we report here.

### Limitations

The pressure difference between left and right ventricles may be one of the factors affecting the mechanical asynchrony between ventricles, but obtaining intraventricular pressure may need invasive examination. For healthy volunteers and some patients with hemodynamic instability, it is difficult to obtain such invasive intraventricular pressure measurement data.

In addition, left ventricular systolic pressure and end diastolic pressure could also affect left and right ventricular synchrony. Finally, the consequent effects of parameters such as blood pressure and E/e 'may merit further research.

## Conclusion

By using Pulse-wave Doppler echocardiographic approaches, we have explored and characterized the cardiac mechanical sequence in LV and RV and their relationships in three large species. Significant variations in LV and RV systolic and diastolic mechanical sequences are demonstrated between healthy subjects and HF patients. Systolic and diastolic ventricular mechanical sequence assessment by echocardiography should be done before and after CRT, as a guide for the optimization of pacing indices. To gain a highly effective therapeutic response, ventricular mechanical sequence should be considered for individual patients and recorded in routine echocardiographic examination.

## Data Availability Statement

The original data upon which this article is based is available from the corresponding author upon reasonable request.

## Ethics Statement

The studies involving human participants were reviewed and approved by the Peking University People's Hospital following approval by the local Ethics Committee. The patients/participants provided their written informed consent to participate in this study. All studies involving animal procedures were approved by the Animal Care and Use Committee of Peking University (IACUC approval numbers: 2011–0010 for canine and IMM-ZhangXQ-1 for primate studies) and complied with the principles of laboratory animal care of the National Academy of Sciences/National Research Council of the People's Republic of China.

## Author Contributions

TZ was responsible for overall study design. ML supervised the canine. XZ supervised the primate experiments. RZ, YZ, WZ, and YL performed the animal experiments. WJ, CY, DL, XQ, LK, and SL recruited and studied the healthy human subjects and heart failure patients. ZW performed data analysis and figure preparation. CH performed data statistical analysis and writing of this article. All authors contributed to the article and approved the submitted version.

## Funding

This study was funded by a National Key Research and Development (R&D) Program of China grant (2018YFA0801405), the Beijing Science and Technology Project (81670090) and the National Natural Science Funding of China (91646202, 81471063, 31221002, and 31871181).

## Conflict of Interest

The authors declare that the research was conducted in the absence of any commercial or financial relationships that could be construed as a potential conflict of interest.

## Publisher's Note

All claims expressed in this article are solely those of the authors and do not necessarily represent those of their affiliated organizations, or those of the publisher, the editors and the reviewers. Any product that may be evaluated in this article, or claim that may be made by its manufacturer, is not guaranteed or endorsed by the publisher.

## Abbreviations

BSA: body surface area
BW: body weight
CRT: cardiac resynchronization therapy
DBP: diastolic blood pressure
FAC: fractional area change
HF: heart failure
HR: heart rate
IVMD: inter-ventricular mechanical delay
IVMDd: diastolic interventricular mechanical delay
IVMDs: systolic interventricular mechanical delay
LV: left ventricle
LVEDV: left ventricular end-diastolic volume
LVEF: left ventricular ejection fraction
LVESV: left ventricular end-systolic volume
LVMDd: left ventricular mechanical delay in diastole
LVMDs: left ventricular mechanical delay in systole
LV_PEP_: left ventricular pre-ejection period
Q-MVe: the time interval from the onset of the QRS complex to the onset of early diastolic E wave of the mitral valve
Q-TVe: the time interval from the onset of QRS complex to the onset of early diastolic E wave of tricuspid valve
RV: right ventricle
RVMDd: right ventricular mechanical delay in diastole
RVMDs: right ventricular mechanical delay in systole
RV_PEP_: right ventricular pre-ejection period
SBP: systolic blood pressure.

## References

[1] Ambrosy AP Fonarow GC Butler J Chioncel O Greene SJ Vaduganathan M. The global health and economic burden of hospitalizations for heart failure: lessons learned from hospitalized heart failure registries. J Am Coll Cardiol. (2014) 63:1123–33. 10.1016/j.jacc.2013.11.053 24491689

[2] Maggioni AP Anker SD Dahlström U Filippatos G Ponikowski P Zannad F. Are hospitalized or ambulatory patients with heart failure treated in accordance with European Society of Cardiology guidelines? Evidence from 12,440 patients of the ESC Heart Failure Long-Term Registry. Eur J Heart Fail. (2013) 15:1173–84. 10.1093/eurjhf/hft134 23978433

[3] Cleland JG Abraham WT Linde C Gold MR Young JB Claude Daubert J. An individual patient meta-analysis of five randomized trials assessing the effects of cardiac resynchronization therapy on morbidity and mortality in patients with symptomatic heart failure. Eur Heart J. (2013) 34:3547–56. 10.1093/eurheartj/eht290 23900696 PMC3855551

[4] Starling RC. The heart failure pandemic: changing patterns, costs, and treatment strategies. Cleve Clin J Med. (1998) 65:351–8. 10.3949/ccjm.65.7.351 9679390

[5] Cleland JGF Daubert J-C Erdmann E Freemantle N Gras D Kappenberger L. The effect of cardiac resynchronization on morbidity and mortality in heart failure. N Engl J Med. (2005) 352:1539–49. 10.1056/nejmoa050496 15753115

[6] Linde C Leclercq C Rex S Garrigue S Lavergne T Cazeau S. Long-term benefits of biventricular pacing in congestive heart failure: results from the MUltisite STimulation in cardiomyopathy (MUSTIC) study. J Am Coll Cardiol. (2002) 40:111–8. 10.1016/s0735-1097(02)01932-0 12103264

[7] Stankovic I Belmans A Prinz C Ciarka A Maria Daraban A Kotrc M. The association of volumetric response and long-term survival after cardiac resynchronization therapy. Eur Heart J Cardiovasc Imaging. (2017) 18:1109–17. 10.1093/ehjci/jex188 28950379

[8] Kirk JA Kass DA. Electromechanical dyssynchrony and resynchronization of the failing heart. Circ Res. (2013) 113:765–76. 10.1161/circresaha.113.300270 23989718 PMC3874431

[9] Haugaa KH Edvardsen T Smiseth OA. Mechanical dyssynchrony-resurrected as a flashing and rocking parameter to predict prognosis after cardiac resynchronization therapy. Eur Heart J Cardiovasc Imaging. (2017) 18:1118–9. 10.1093/ehjci/jex198 28984891

[10] Kuznetsov VA Soldatova AM Kasprzak JD Krinochkin DV Melnikov NN. Echocardiographic markers of dyssynchrony as predictors of super-response to cardiac resynchronisation therapy - a pilot study. Cardiovasc Ultrasound. (2018) 16:24. 10.1186/s12947-018-0140-0 30285762 PMC6167795

[11] Zhu TG Patel C Martin S Quan X Wu Y Burke JF. Ventricular transmural repolarization sequence: its relationship with ventricular relaxation and role in ventricular diastolic function. Eur Heart J. (2009) 30:372–80. 10.1093/eurheartj/ehn585 19147608

[12] Boukens BJ Walton R Meijborg VM Coronel R. Transmural electrophysiological heterogeneity, the T-wave and ventricular arrhythmias. Prog Biophys Mol Biol. (2016) 122:202–14. 10.1016/j.pbiomolbio.2016.05.009 27221779

[13] Zhang Z Letsas KP Yang Y Korantzopoulos P Li G Yan GX. Notching early repolarization pattern in inferior leads increases risk of ventricular tachyarrhythmias in patients with acute myocardial infarction: a meta-analysis. Sci Rep. (2015) 5:15845. 10.1038/srep15845 26521690 PMC4629141

[14] Galderisi M Cattaneo F Mondillo S. Doppler echocardiography and myocardial dyssynchrony: a practical update of old and new ultrasound technologies. Cardiovasc Ultrasound. (2007) 5:28. 10.1186/1476-7120-5-28 17822551 PMC2034540

[15] AlJaroudi W Alraies MC Hachamovitch R Jaber WA Brunken R Cerqueira MD. Association of left ventricular mechanical dyssynchrony with survival benefit from revascularization: a study of gated positron emission tomography in patients with ischemic LV dysfunction and narrow QRS. Eur J Nucl Med Mol Imaging. (2012) 39:1581–91. 10.1007/s00259-012-2171-3 22699531

[16] Kano N Okumura T Isobe S Sawamura A Watanabe N Fukaya K. Left ventricular phase entropy: Novel prognostic predictor in patients with dilated cardiomyopathy and narrow QRS. J Nucl Cardiol. (2018) 25:1677–87. 10.1007/s12350-017-0807-1 28176257

[17] Shin SH Hung CL Uno H Hassanein AH Verma A Bourgoun M. Mechanical dyssynchrony after myocardial infarction in patients with left ventricular dysfunction, heart failure, or both. Circulation. (2010) 121:1096–103. 10.1161/circulationaha.109.863795 20176989

[18] Fudim M Fathallah M Shaw LK James O Samad Z Piccini JP. The prognostic value of diastolic and systolic mechanical left ventricular dyssynchrony among patients with coronary artery disease and heart failure. J Nucl Cardiol. (2020) 27:1622–32. 10.1007/s12350-019-01843-4 31392509

[19] Stegemann B Dreger H Ismer B Baumann G Melzer C. Left ventricular asynchrony in patients with right bundle branch block and normal ejection fraction. Pacing Clin Electrophysiol. (2013) 36:63–8. 10.1111/pace.12043 23121169

[20] Yu C-M Lin H Ho P-C Yang H. Assessment of left and right ventricular systolic and diastolic synchronicity in normal subjects by tissue Doppler echocardiography and the effects of age and heart rate. Echocardiography. (2003) 20:19–27. 10.1046/j.1540-8175.2003.00003.x 12848694

[21] Quan X Zhu T-G Guo S Ma J-X Wang X Guo J-H. Ventricular synchronicity: observations comparing pulse flow and tissue Doppler assessment in a Chinese healthy adult cohort. Chin Med J (Engl). (2012) 125:27–32. 10.3760/cma.j.issn.0366-6999.2012.01.006 22340461

[22] Grines CL Bashore TM Boudoulas H Olson S Shafer P Wooley CF. Functional abnormalities in isolated left bundle branch block. The effect of interventricular asynchrony. Circulation. (1989) 79:845–53. 10.1161/01.cir.79.4.845 2924415

[23] Azazy AS Soliman M Yaseen R Mena M Sakr H. Left ventricular dyssynchrony assessment using tissue synchronization imaging in acute myocardial infarction. Avicenna J Med. (2019) 9:48–54. 10.4103/ajm.AJM_168_18 31143697 PMC6530268

[24] Lang RM Badano LP Mor-Avi V Afilalo J Armstrong A Ernande L. Recommendations for cardiac chamber quantification by echocardiography in adults: an update from the American Society of Echocardiography and the European Association of Cardiovascular Imaging. Eur Heart J Cardiovasc Imaging. (2015) 16:233–70. 10.1093/ehjci/jev014 25712077

[25] Yan GX Rials SJ Wu Y Liu T Xu X Marinchak RA. Ventricular hypertrophy amplifies transmural repolarization dispersion and induces early afterdepolarization. Am J Physiol Heart Circ Physiol. (2001) 281:H1968–1975. 10.1152/ajpheart.2001.281.5.h1968 11668057

[26] Yan GX Shimizu W Antzelevitch C. Characteristics and distribution of M cells in arterially perfused canine left ventricular wedge preparations. Circulation. (1998) 98:1921–7. 9799214 10.1161/01.cir.98.18.1921

[27] Yu CM Lin H Zhang Q Sanderson JE. High prevalence of left ventricular systolic and diastolic asynchrony in patients with congestive heart failure and normal QRS duration. Heart. (2003) 89:54–60. 10.1136/heart.89.1.54 12482792 PMC1767510

[28] Bleeker GB Schalij MJ Molhoek SG Holman ER Verwey HF Steendijk P. Frequency of left ventricular dyssynchrony in patients with heart failure and a narrow QRS complex. Am J Cardiol. (2005) 95:140–2. 10.1016/j.amjcard.2004.08.082 15619414

[29] Yusuf J Agrawal DK Mukhopadhyay S Mehta V Trehan V Tyagi S. Fragmented narrow QRS complex: predictor of left ventricular dyssynchrony in non-ischemic dilated cardiomyopathy. Indian Heart J. (2013) 65:172–9. 10.1016/j.ihj.2013.02.007 23647897 PMC3860618

[30] Naqvi TZ Rafique AM Peter CT. Echo-driven V-V optimization determines clinical improvement in non responders to cardiac resynchronization treatment. Cardiovasc Ultrasound. (2006) 4:39. 10.1186/1476-7120-4-39 17049099 PMC1636667

