# Supplementary material for: A Comparative Study of Systolic and Diastolic Mechanical Synchrony in Canine, Primate, and Healthy and Failing Human Hearts
Source: Front Cardiovasc Med. 2021 Oct 28;8:750067. doi: 10.3389/fcvm.2021.750067 (PMC8581184; doi:10.3389/fcvm.2021.750067)
Supplement: Supplementary file 1 [file Table_1.docx]

**Supplementary materials**

Supplemental Table 1. Binary logistic regression analysis of ventricular mechanical sequence in systole

|  |  |  | **Age** | **Gender** | **BSA** | **HR** | **Constant** |
| --- | --- | --- | --- | --- | --- | --- | --- |
| **β** | | **Monkeys(n=59)** | **-0.236** | **-** | **-** | **-0.129** | **4.873** |
|  |  | **Canines(n=15)** | **-0.028** | **-** | **-** | **-0.036** | **3.476** |
|  |  | **Normal(n=100)** | **-0.015** | **0.556** | **-0.045** | **-0.035** | **3.158** |
|  |  | **HF(n=39)** | **-0.019** | **0.632** | **-0.036** | **-0.041** | **3.327** |
| **S.E** | | **Monkeys(n=59)** | **0.018** | **-** | **-** | **0.032** | **5.342** |
|  |  | **Canines(n=15)** | **0.026** | **-** | **-** | **0.013** | **3.498** |
|  |  | **Normal(n=100)** | **0.033** | **0.665** | **1.662** | **0.029** | **4.706** |
|  |  | **HF(n=39)** | **0.038** | **0.673** | **1.595** | **0.027** | **4.329** |
| **Wals** | | **Monkeys(n=59)** | **0.272** | **-** | **-** | **1.422** | **0.372** |
|  |  | **Canines(n=15)** | **0.345** | **-** | **-** | **1.387** | **0.564** |
|  |  | **Normal(n=100)** | **0.211** | **0.699** | **0.001** | **1.513** | **0.451** |
|  |  | **HF(n=39)** | **0.256** | **0.787** | **0.002** | **1.238** | **0.372** |
| ***p* Value** | | **Monkeys(n=59)** | **0.539** | **-** | **-** | **0.083** | **0.89** |
|  |  | **Canines(n=15)** | **0.478** | **-** | **-** | **0.056** | **0.383** |
|  |  | **Normal(n=100)** | **0.646** | **0.403** | **0.979** | **0.219** | **0.502** |
|  |  | **HF(n=39)** | **0.823** | **0.484** | **0.934** | **0.125** | **25.37** |
| **OR** | | **Monkeys(n=59)** | **0.932** | **-** | **-** | **0.856** | **32.27** |
|  |  | **Canines(n=15)** | **0.946** | **-** | **-** | **0.978** | **26.785** |
|  |  | **Normal(n=100)** | **0.985** | **1.744** | **0.956** | **0.965** | **23.526** |
|  |  | **HF(n=39)** | **0.836** | **1.657** | **0.837** | **0.783** | **21.783** |
| **OR 95% CI** | **lower limit** | **Monkeys(n=59)** | **0.842** | **-** | **-** | **0.723** | **-** |
|  |  | **Canines(n=15)** | **0.246** | **-** | **-** | **0.932** | **-** |
|  |  | **Normal(n=100)** | **0.924** | **0.474** | **0.037** | **0.912** | **-** |
|  |  | **HF(n=39)** | **0.783** | **0.537** | **0.024** | **0.653** | **-** |
|  | **upper limit** | **Monkeys(n=59)** | **1.236** | **-** | **-** | **1.234** | **-** |
|  |  | **Canines(n=15)** | **1.403** | **-** | **-** | **1.024** | **-** |
|  |  | **Normal(n=100)** | **1.051** | **6.426** | **24.867** | **1.021** | **-** |
|  |  | **HF(n=39)** | **1.278** | **8.903** | **46.783** | **1.402** | **-** |

BSA, body surface area ; HR, heart rate, S.E, standard error

Supplemental Table 2. Binary logistic regression analysis of ventricular mechanical sequence in diastole

|  |  |  | **Age** | **Gender** | **BSA** | **HR** | **Constant** |
| --- | --- | --- | --- | --- | --- | --- | --- |
| **β** | | **Monkeys(n=59)** | **-0.224** | **-** | **-** | **-0.123** | **4.629** |
|  |  | **Canines(n=15)** | **-0.027** | **-** | **-** | **-0.034** | **3.302** |
|  |  | **Normal(n=100)** | **-0.014** | **0.528** | **-0.043** | **-0.033** | **3.000** |
|  |  | **HF(n=39)** | **-0.018** | **0.600** | **-0.034** | **-0.039** | **3.161** |
| **S.E** | | **Monkeys(n=59)** | **0.017** | **-** | **-** | **0.030** | **5.075** |
|  |  | **Canines(n=15)** | **0.025** | **-** | **-** | **0.012** | **3.323** |
|  |  | **Normal(n=100)** | **0.031** | **0.632** | **1.579** | **0.028** | **4.471** |
|  |  | **HF(n=39)** | **0.036** | **0.639** | **1.515** | **0.026** | **4.113** |
| **Wals** | | **Monkeys(n=59)** | **0.258** | **-** | **-** | **1.351** | **0.353** |
|  |  | **Canines(n=15)** | **0.328** | **-** | **-** | **1.318** | **0.536** |
|  |  | **Normal(n=100)** | **0.200** | **0.664** | **0.001** | **1.437** | **0.428** |
|  |  | **HF(n=39)** | **0.243** | **0.748** | **0.002** | **1.176** | **0.353** |
| ***p* Value** | | **Monkeys(n=59)** | **0.512** | **-** | **-** | **0.079** | **0.846** |
|  |  | **Canines(n=15)** | **0.454** | **-** | **-** | **0.053** | **0.364** |
|  |  | **Normal(n=100)** | **0.614** | **0.383** | **0.930** | **0.208** | **0.477** |
|  |  | **HF(n=39)** | **0.782** | **0.460** | **0.887** | **0.119** | **24.102** |
| **OR** | | **Monkeys(n=59)** | **0.885** | **-** | **-** | **0.813** | **30.657** |
|  |  | **Canines(n=15)** | **0.899** | **-** | **-** | **0.929** | **25.446** |
|  |  | **Normal(n=100)** | **0.936** | **1.657** | **0.908** | **0.917** | **22.350** |
|  |  | **HF(n=39)** | **0.794** | **1.574** | **0.795** | **0.744** | **20.694** |
| **OR 95% CI** | **lower limit** | **Monkeys(n=59)** | **0.800** | **-** | **-** | **0.687** | **-** |
|  |  | **Canines(n=15)** | **0.234** | **-** | **-** | **0.885** | **-** |
|  |  | **Normal(n=100)** | **0.878** | **0.450** | **0.035** | **0.866** | **-** |
|  |  | **HF(n=39)** | **0.744** | **0.510** | **0.023** | **0.620** | **-** |
|  | **upper limit** | **Monkeys(n=59)** | **1.174** | **-** | **-** | **1.172** | **-** |
|  |  | **Canines(n=15)** | **1.333** | **-** | **-** | **0.973** | **-** |
|  |  | **Normal(n=100)** | **0.998** | **6.105** | **23.624** | **0.970** | **-** |
|  |  | **HF(n=39)** | **1.214** | **8.458** | **44.444** | **1.332** | **-** |

BSA,body surface area ; HR, heart rate；S.E,
